# Therapeutic Potential of Selectively Targeting the α_2C_-Adrenoceptor in Cognition, Depression, and Schizophrenia—New Developments and Future Perspective

**DOI:** 10.3389/fpsyt.2017.00144

**Published:** 2017-08-14

**Authors:** Madeleine Monique Uys, Mohammed Shahid, Brian Herbert Harvey

**Affiliations:** ^1^Division of Pharmacology, Centre of Excellence for Pharmaceutical Sciences, North-West University, Potchefstroom, South Africa; ^2^Orion Pharma, Orion Corporation, Nottingham, United Kingdom

**Keywords:** Alzheimer’s disease, α_2C_-antagonism, schizophrenia, depression, cognition, ORM-10921

## Abstract

α_2A_- and α_2C_-adrenoceptors (ARs) are the primary α_2_-AR subtypes involved in central nervous system (CNS) function. These receptors are implicated in the pathophysiology of psychiatric illness, particularly those associated with affective, psychotic, and cognitive symptoms. Indeed, non-selective α_2_-AR blockade is proposed to contribute toward antidepressant (e.g., mirtazapine) and atypical antipsychotic (e.g., clozapine) drug action. Both α_2C_- and α_2A_-AR share autoreceptor functions to exert negative feedback control on noradrenaline (NA) release, with α_2C_-AR heteroreceptors regulating non-noradrenergic transmission (e.g., serotonin, dopamine). While the α_2A_-AR is widely distributed throughout the CNS, α_2C_-AR expression is more restricted, suggesting the possibility of significant differences in how these two receptor subtypes modulate regional neurotransmission. However, the α_2C_-AR plays a more prominent role during states of low endogenous NA activity, while the α_2A_-AR is relatively more engaged during states of high noradrenergic tone. Although augmentation of conventional antidepressant and antipsychotic therapy with non-selective α_2_-AR antagonists may improve therapeutic outcome, animal studies report distinct yet often opposing roles for the α_2A_- and α_2C_-ARs on behavioral markers of mood and cognition, implying that non-selective α_2_-AR antagonism may compromise therapeutic utility both in terms of efficacy and side-effect liability. Recently, several highly selective α_2C_-AR antagonists have been identified that have allowed deeper investigation into the function and utility of the α_2C_-AR. ORM-13070 is a useful positron emission tomography ligand, ORM-10921 has demonstrated antipsychotic, antidepressant, and pro-cognitive actions in animals, while ORM-12741 is in clinical development for the treatment of cognitive dysfunction and neuropsychiatric symptoms in Alzheimer’s disease. This review will emphasize the importance and relevance of the α_2C_-AR as a neuropsychiatric drug target in major depression, schizophrenia, and associated cognitive deficits. In addition, we will present new prospects and future directions of investigation.

## Introduction

The α_2_-adrenoceptor (AR) plays an important role in modulating the release of noradrenaline (NA) and various other important neurotransmitters in the central nervous system (CNS), providing a solid construct why drugs that target these receptors have clinical utility in several major neuropsychiatric disorders (1). The α_2_- (and α_1_-) AR plays a prominent role in the functioning of the prefrontal cortex (PFC) and as such mediates the effect of normal, aroused, and stressed NA levels on memory and other cognitive processes (2). To this end α_2_-AR antagonists mianserin and mirtazapine have seen widespread use in the therapy of major depressive disorder (MDD), while almost all atypical antipsychotics display moderate to potent levels of α_2_-AR antagonism, which has been suggested to underlie the atypical profile of antipsychotics such as clozapine, quetiapine, risperidone, and asenapine (3, 4). Importantly, both conventional antipsychotics (5–7) and antidepressants (8–10) show improved efficacy following augmentation with an α_2_-AR antagonist. Furthermore, cognitive parameters are also influenced by α_2_-AR modulation with α_2_-AR antagonism shown to improve attentional, verbal, and episodic memory deficits in patients with frontal dementia, although spatial working memory is unaffected (11). This is because stimulation of the cortical postsynaptic α_2A_-AR by NA is critical in the function of specific cognitive domains such as working memory (12), which is why α_2_-AR agonists are successfully used in the treatment of cognitive aspects of attention-deficit hyperactivity disorder (ADHD) (13). However, studies have indicated that α_2_-AR subtypes may not equally contribute to these beneficial effects on mood, psychotic, and cognitive disorders. In fact, findings from transgenic mouse studies have indicated distinct and sometimes opposing roles for the α_2A_-AR and α_2C_-AR (14–16), the two primary α_2_-AR subtypes involved in the regulation of CNS neurotransmission (refer to Table 1 for summary). Before the availability of sufficiently subtype-selective ligands, evidence from transgenic mouse studies have indicated a potential therapeutic role for selective antagonism of the α_2C_-AR in MDD, schizophrenia and associated cognitive impairment (16). More recently, the availability of highly selective α_2C_-AR antagonists for use in preclinical research has produced evidence confirming the antipsychotic-like, antidepressant-like, and pro-cognitive effects of this treatment strategy in animal models of schizophrenia and MDD (17–21). Genetic studies have also highlighted the potential involvement of the α_2C_-AR in these neuropsychiatric illnesses, with evidence that genetic polymorphism of the α_2C_-AR is associated with dysfunction in MDD (22), ADHD (23), and schizophrenia (24). With the first highly selective α_2C_-AR subtype antagonist, ORM-12741, showing improvement of cognitive parameters in Alzheimer’s Disease in Phase IIa clinical trials (25) and against a back-drop of evidence from transgenic mouse and other translational rodent models, the potential therapeutic benefit of selectively blocking α_2C_-ARs for the treatment of cognitive dysfunction in mood and psychotic disorders has attracted renewed interest. This review will summarize evidence from transgenic mouse models relating to the function of the α_2C_-AR in related neuropsychiatric function as well as present studies reporting on the therapeutic efficacy of selective α_2C_-AR antagonists in illness-specific models of MDD and schizophrenia in rats. Following a short overview of the functional roles for α_2A_ and α_2C_-ARs, we will outline reasons for renewed interest in selective α_2C_-AR antagonism as a therapeutic target, its role in neurotransmitter regulation, and the evidence base for targeting this receptor for treating MDD and schizophrenia. We close with a brief discussion on the potential therapeutic benefits for α_2C_-AR modulation in other neuropsychiatric disorders and highlight progress in developing α_2C_-AR-related tools and technology to facilitate future basic and clinical research.

**Table 1:** Summary of opposing effects mediated through the α_2C_-AR and the α_2A_-AR.

Parameter	α_2C_	α_2A_	Reference
CNS distribution	10% of α_2_-ARs in CNS	90% of α_2_-ARs in CNS	(26–28)
	Located primarily in the striatum, hippocampus, olfactory tubercle, cortex	Widely spread throughout CNS structures	

NA	NA has higher affinity and potency for α_2C_-AR	NA has lower affinity and potency for α_2A_-AR	(29–30, 54)
	Slower deactivation upon removal of NA	Faster deactivation upon removal of NA	
	Slow presynaptic negative feedback at low endogenous NA concentrations (10–100 nM)	Fast presynaptic negative feedback at high endogenous NA concentrations (0.1–10 μM)	
	Receptor density is regulated by the synaptic availability of NA	Receptor density is not regulated by the availability of NA	

5-HT	Modulates 5-HT synthesis to lesser extent than α_2A_-AR	Main modulator of 5-HT synthesis	(31–33)
	Inhibits 5-HT release to a lesser extent than α_2A_-AR	Main inhibitor of 5-HT release	

DOPA	Antagonism increases and agonism decreases synthesis *via* feedback inhibition on tyrosine hydroxylase	Neither agonism nor antagonism affects DOPA levels	(31)

Cognitive parameters	α_2C_-AR antagonism improves spatial and working memory	α_2A_-AR agonism improves spatial and working memory; enhances cognition	(34–39)

Antidepressant activity	α_2C_-AR activation increases immobility in the FST	α_2A_-AR antagonism increases immobility and insensitivity to the effects of tricyclic antidepressants in the FST	(17, 18, 21, 40–42)
	α_2C_-AR deactivation decreases immobility in the FST		

Antipsychotic activity	α_2C_-AR-agonism improves deficits in PPI in transgenic α_2C_-OE mice	α_2A_-AR antagonism does not improve PPI deficits	(17–20, 43, 44)
	Selective α_2C_-AR antagonists improve PPI deficit in other rodent models		

*AR, adrenoceptor; DA, dopamine; DOPA, 3,4-dihydroxyphenylalanine; CNS, central nervous system; NA, noradrenaline; 5-HT, serotonin; FST, forced swim test; KO, receptor knockout; OE, receptor overexpression; PPI, prepulse inhibition test*.

## Distinct Roles for α_2_-AR Subtypes

The α_2_-AR is a member of the G-protein-coupled receptor (GPCR) superfamily, belonging to the rhodopsin-like or Class A GPCR receptors (45). α_2_-ARs couple to heterotrimeric G_i/o_ proteins when activated by their endogenous agonist, leading to inhibition of adenylyl cyclase and voltage-gated calcium channels, and activation of mitogen-activated protein kinase signaling cascades (15, 46). In the CNS, GPCRs and ion channels are targeted to the membrane of dendritic postsynaptic terminals in and around the postsynaptic density (PSD) *via* interaction with various scaffolding proteins (45). These proteins function as adaptors, regulators, and effectors of postsynaptic signaling to enable neural transmission and biological response. Spinophilin in particular is associated with the α_2_-AR (45), the relevance of which will be discussed later.

The presynaptic α_2_-AR autoreceptor inhibits NA synthesis and release and as such plays an important role in negative feedback, while presynaptic α_2_-AR heteroreceptors located on dopaminergic, serotoninergic, glutamatergic, and other terminals regulate the release of these latter transmitters (15, 46). Postsynaptic activation of α_2_-ARs in turn modulates neuronal excitability *via* regulation of ion channels, including the direct modulation of inwardly rectifying potassium channels and the indirect modulation of hyperpolarization-activated channels (46). While presynaptic action at α_2_-ARs affect neuropsychiatric processes through a cascade of effects on neurotransmitter feedback and regulation, postsynaptic activation of α_2_-ARs, specifically the α_2A_-AR, is associated with critical regulation and strengthening of working memory (12). Indeed, prefrontal cortical networks regulating various aspects of attention, cognition, and emotion require optimal catecholamine signaling, including stimulation of postsynaptic α_2_-ARs by NA to regulate “top-down” control of the PFC over subcortical regions (12, 47). This explains, for example, why α_2_-AR agonists favoring the α_2A_-AR have beneficial effects on memory and cognition in ADHD. However, α_2_-AR-mediated regulation of CNS function extends to the peripheral nervous system too. In this regard, the gut microbiome is increasingly being seen as a causal factor in psychiatric illness (48). Gut status is enabled to signal the CNS *via* a number of monoaminergic receptors located in the enteric nervous system (48), in particular dopamine (DA) (D_2_), serotonin (5-HT_3_; 5-HT_4_), and NA receptors, the latter *via* inhibition of vagal (parasympathetic) activity through presynaptic α_2_ receptors (49). Notwithstanding the neurophysiological importance of postsynaptic α_2_-AR activation, the literature increasingly points to selectively targeting specific α_2_-AR subtypes to exert control over presynaptic modulation of various neurotransmitter feedback systems associated with cognitive and affective functioning. While α_2_-ARs are collectively important in neural transmission, this review will delineate the therapeutic effects associated with modulation of the presynaptic α_2C_-AR.

The presynaptic α_2_-AR consists of three subtypes which are conserved across mammalian species, identified as the α_2A/D_, α_2B_, and α_2C_-AR-subtypes; the α_2A/D_ designation refers to a small difference in amino acid sequence in rodents (α_2D_) as opposed to that in humans and rabbits (α_2A_) (50, 51). The rodent α_2D_-AR, however, is presumed to reflect the same physiological processes and pharmacological outcomes as the α_2A_-AR, and studies on this receptor in rodents is, therefore, reported as findings for the α_2A_-AR. The α_2_-AR subtypes have dissimilar tissue distribution patterns, along with distinct physiological and pharmacological profiles (51, 52). While all three receptors are present in the CNS, the α_2B_ receptor is mainly expressed in the thalamus and does not seem to contribute to CNS auto- and heteroreceptor function (53). The α_2A_-ARs and α_2C_-ARs, on the other hand, are the primary α_2_-ARs modulating neurotransmission in the CNS (33, 53, 54), with the α_2C_-AR recognized to play a very distinct and specific role in memory, cognition, and mood disorders in a manner different to that of the α_2A_-AR. These separate effects will become evident in this review, and are summarized in Table 1.

Although 90% of α_2_-ARs in the CNS are contributed by the α_2A_-AR, the expression of the α_2C_-AR is more discrete, constituting approximately 10% of the total (26). Nevertheless, the α_2C_-AR seems to play a very important role in neurotransmission and potentially in the dysregulation observed in neuropsychiatric illness. Thus α_2C_-ARs densely populate the ventral and dorsal striatum and the hippocampus in humans (27, 51, 55), monkeys, and rodents (56). Dense population in the olfactory tubercle is also evident, while more subtle cortical expression is also evident (27, 28). The cerebellum is devoid of these receptors. Importantly, these same brain areas are populated by the α_2A_-AR, among others (27, 57, 58). The specific distribution pattern for the α_2C_-AR asserts its role in illnesses involving hippocampal and striatal dysfunction, such as schizophrenia and MDD, and in conditions characterized by cognitive deficits and cognitive decline involving these cortico-limbic structures (e.g., Alzheimer’s disease) (27, 59–61).

The distribution of α_2C_-ARs in human, monkey, and rodent brains are analogous (55, 56, 59, 62), implying that neuropharmacological data from transgenic mouse models and from rodent animal models may be relevant for humans also. Due to the paucity of sufficiently subtype-selective ligands, of which only a few have become available for preclinical investigation during the last decade (17–19, 63), transgenic mouse models have predominantly been used in earlier work to shed light on the physiology and pharmacology of the different α_2_-AR subtypes. Transgenic mouse models employ targeted genetic deletion or overexpression of the α_2A_-AR and/or α_2C_-AR to examine consequence of loss or gain of receptor function, respectively (16). Findings from these transgenic mouse models have suggested distinct and often seemingly opposing CNS roles for the α_2A_-AR and α_2C_-AR, with the implication that non-selective α_2_-AR modulation might potentially negate beneficial effects which could be attained by subtype-selective targeting.

Studies in genetically modified mouse models predicting antipsychotic-, antidepressant-, and pro-cognitive-like effects has brought to light an important role for the α_2C_-AR, as illustrated by a modulation of behavior and neurotransmission akin to that seen in neuropsychiatric disorders like MDD, schizophrenia, and their associated cognitive deficits (16, 40, 43, 64–67). However, transgenic mouse studies may suffer from the unknown contribution by physiological compensatory changes that take place as a consequence of lifelong absence or overexpression of α_2_-ARs (17). For example, Sallinen et al. (43) demonstrated deficient sensorimotor gating (see Cognitive Deficits Associated With MDD and Schizophrenia) in α_2C_-KO mice, suggesting that α_2C_-AR antagonism may induce effects likened to psychotomimetic agents such as phencyclidine (PCP). This contradicts recent findings described in the social isolation rearing (SIR) and *N*-methyl-d-aspartate (NMDA)-antagonist models of schizophrenia where selective α_2C_-AR antagonists, *improved* sensorimotor gating deficits (18, 20). This type of anomaly underscores the necessity to verify results obtained using transgenic mouse models with studies employing selective α_2C_-AR ligands in more naturalistic animal models with good validity for the chosen human disorder.

The next section discusses findings regarding the role of the α_2C_-AR as auto- and heteroreceptor in regulating neurotransmitters implicated in depressive and psychotic disorders. The findings from early studies in transgenic mouse models and studies using moderately selective α_2_-AR subtype ligands are reported and are aligned with new evidence using novel highly subtype-selective ligands, where available.

## Role of the α_2C_-AR in Regulating Key Neurotransmitters

Despite a number of new theories that have been put forward to explain the underlying biology and development of mood and psychotic disorders, targeting monoaminergic transmission as a construct toward understanding and treating these disorders remains a relevant subject of investigation [reviewed in Ref. (68)]. The latter review emphasizes that while oxidative stress, neuroinflammation and neuroplastic/degenerative events are implicated in these disorders, selectively and appropriately targeting monoaminergic processes remains a core construct in novel antidepressant and antipsychotic drug development. The α_2C_-AR is associated with various effects on monoamine turnover. When treated with the subtype non-selective α_2_-AR agonist, dexmedetomidine, agonist-induced decreases in monoamine levels were absent in α_2C_-OE mice, while concentrations of DA, NA, and serotonin (5-HT) were shown to be increased in the brains of α_2C_-KO mice (67). Deactivation of α_2C_-ARs might thus facilitate increased CNS monoamine levels, which could be of benefit in disorders where monoamine dysfunction is apparent. However, α_2C_-heteroreceptors modulate other neurotransmitters implicated in the pathophysiology of these disorders, such as γ-aminobutyric acid (GABA), glutamate, and acetylcholine, as will be discussed.

### Noradrenaline

**Figure 1:**
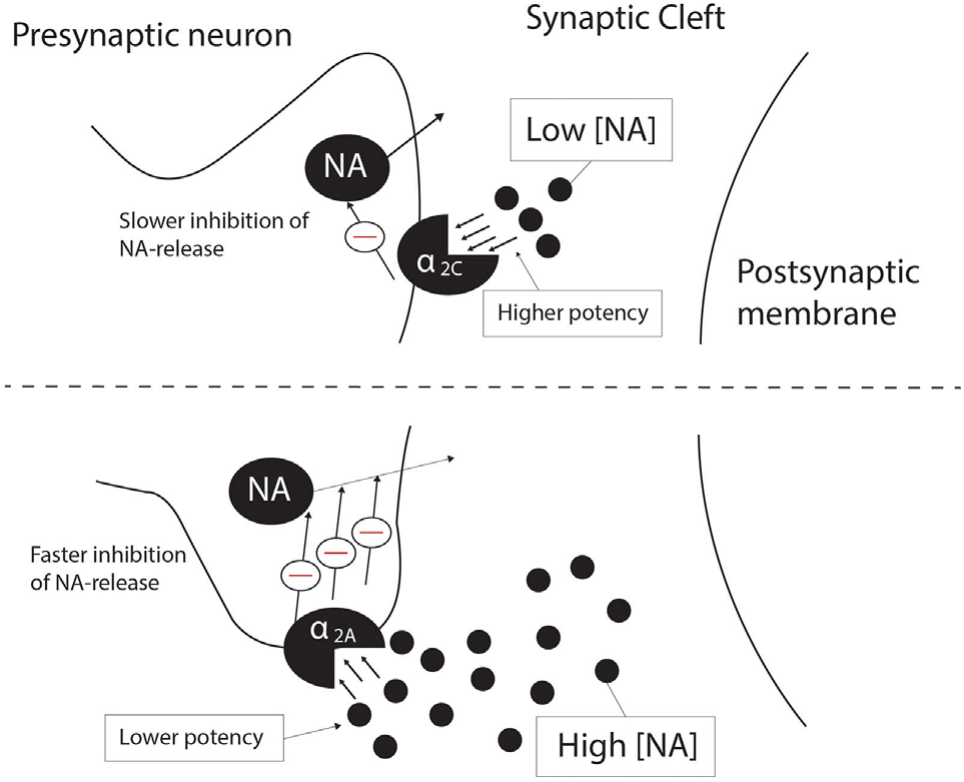
Differential presynaptic inhibition of NA release by the α_2C_-AR (top panel) and the α_2A_-AR (bottom panel). At low endogenous NA concentrations (10–100 nM), the α_2C_-AR is responsible for inhibition of NA release, while the α_2A_-AR inhibits NA release at high endogenous NA concentrations (0.1–10 μM). α_2C_-AR-mediated inhibition of NA release is a slower process than that of α_2A_-AR-mediated inhibition, although the potency and affinity of NA is higher at the α_2C_-AR than at the α_2A_-AR. See text for more detail. NA, noradrenaline; Θ, inhibition.

The α_2A_-AR and α_2C_-AR are the main autoreceptors involved in presynaptic feedback inhibition of NA, with the α_2B_-AR making no significant contribution to NA feedback inhibition (14). However, the potency and affinity of NA at the α_2C_-AR is higher than that for the α_2A_-AR (14, 29, 69), and evidence from peripheral and CNS tissue demonstrates that the α_2C_-AR would inhibit NA release at low [10–100 nM, adapted from Ref. (14)] endogenous concentrations of NA as opposed to high [0.1 –10 μM, adapted from Ref. (14)] concentrations for the α_2A_-AR (14, 26). Deactivation kinetics also differs for the α_2A_-AR and α_2C_-AR, with the α_2C_-AR displaying much slower deactivation upon removal of NA than the α_2A_-AR (29). Despite their more modest presentation in the CNS, α_2C_-ARs will, therefore, have distinct effects on a number of important neurotransmitters (see below), while its effects on NA’ergic transmission cannot be underestimated. Along with the α_2A_-AR, α_2C_-ARs are involved in the presynaptic negative feedback loop on NA release in the cortex, although α_2C_-AR-mediated presynaptic inhibition occurs more slowly than that mediated by α_2A_-ARs (26). Figure 1 depicts this proposed differential regulation on NA feedback and receptor pharmacodynamics mediated by α_2A_-ARs and α_2C_-ARs. Furthermore, the α_2C_-AR produces a limited inhibition of NA release (maximum 20–30% in hippocampal tissue) in contrast to the α_2A_-AR (26), which would suggest that from a therapeutic perspective, α_2C_-AR modulation would provide a more subtle and targeted effect on NA release, while limited effects on NA release could potentially dampen the potential for cardiovascular side effects, which are a significant concern with α_2A_-AR antagonism (26). Ordway and co-workers demonstrated that the density of α_2C_-AR binding sites increases 3 weeks after the destruction of NA terminals in the rodent cerebral cortex, which suggests that α_2C_-AR density is regulated by the synaptic availability of NA. In contrast, altered α_2A_-AR density was not observed under the same conditions (30). This effect of synaptic availability on α_2C_-AR expression might imply a unique role for the α_2C_-AR in disorders characterized by noradrenergic dysregulation, such as MDD and schizophrenia.

The α_2C_-AR has also been implicated in α_2_-autoreceptor-mediated modulation of hippocampal and cortical DA and NA synthesis *via* feedback inhibition on tyrosine hydroxylase, which converts tyrosine to the DA precursor 3,4-dihydroxyphenylalanine (DOPA) (31). These authors used early subtype-specific antagonists and agonists to measure levels of DOPA and NA in rodent hippocampus and cerebral cortex, with α_2B/C_-AR antagonists increasing synthesis of DOPA and α_2B/C_-AR agonists decreasing its synthesis. Although the ligands used in this study were α_2B/C_-AR specific ligands, the expression of α_2B_-ARs is limited to the hypothalamus and does not seem to contribute to auto- and heteroreceptor function in the CNS (53). This study also reported that α_2A_-AR specific antagonism and agonism were devoid of effects on DOPA. However, a limitation of this study is that the subtype-specific ligands used also present with some antagonist activity at 5-HT_1A_ receptors (32). α_2C_-AR selective antagonism could, however, play a role in increasing DA and NA levels and thus be of benefit in the treatment of neuropsychiatric illness. Nevertheless, these findings need to be confirmed using novel, highly subtype-selective α_2_-AR ligands.

### Dopamine

**Figure 2:**
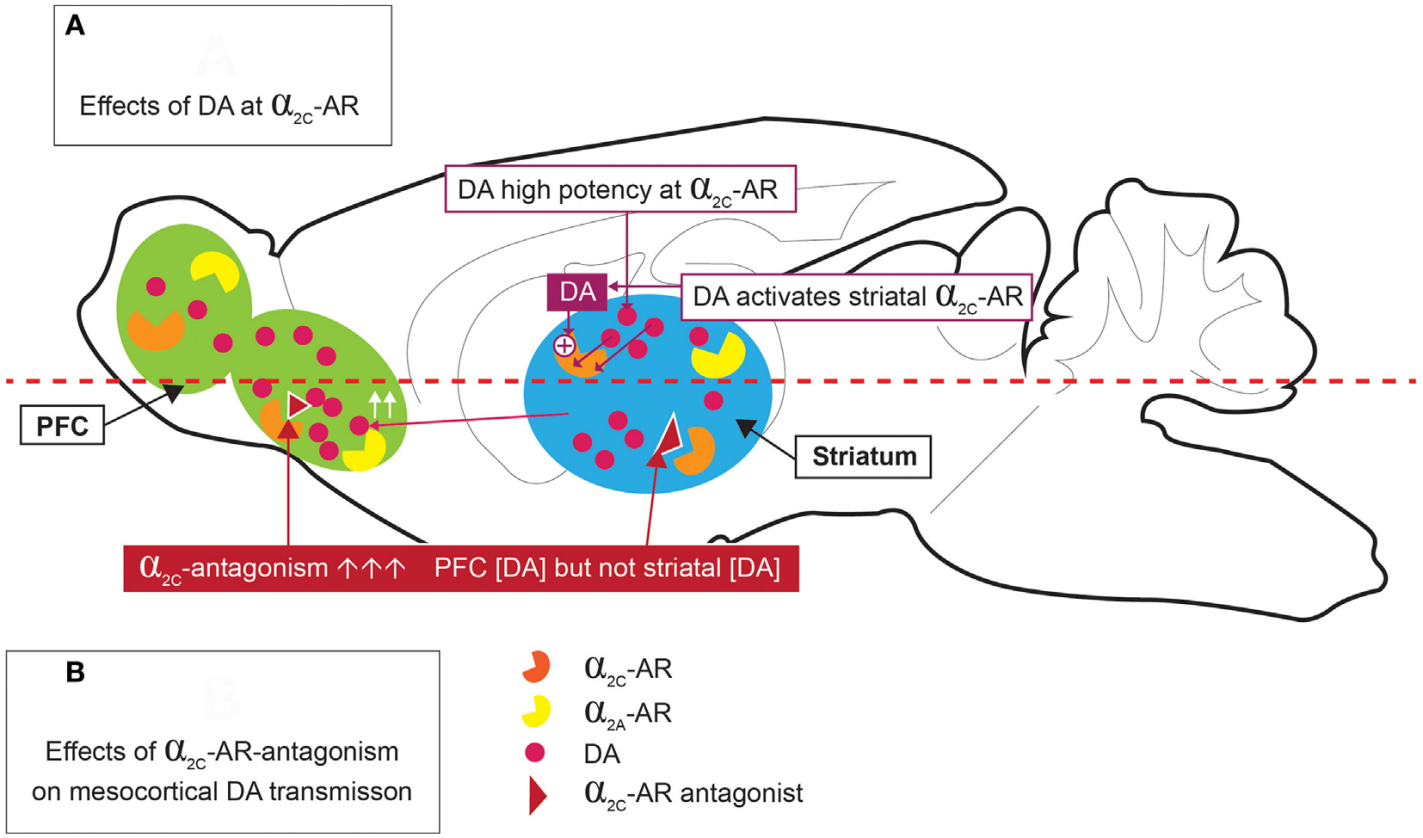
Dopamine (DA) stimulation of α_2C_-ARs [**(A)** top panel], and effects of α_2C_-AR-antagonism on mesocortical DA [**(B)** bottom panel]. DA is a high potency agonist at the α_2C_-AR, where it may have significant implications for DA release in the striatum and prefrontal cortex (PFC). α_2C_-AR antagonism increases PFC DA levels, but not striatal DA levels.

**Figure 3:**
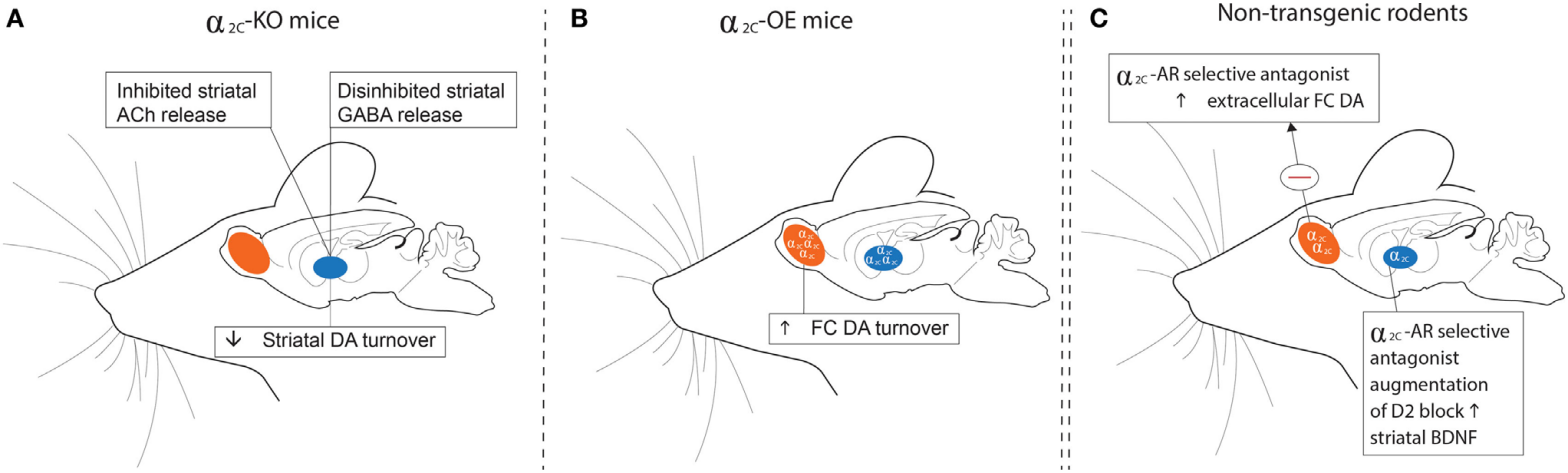
Schematic outline of findings relating to dopaminergic, GABAergic, and cholinergic transmission in the striata and frontal cortices of α_2C_-KO (left panel) and α_2C_-OE (center panel) mice and in non-transgenic rodents (right panel) treated with a selective α_2C_-AR antagonist. **(A)** HVA levels are decreased in the striata of α_2C_-KO mice, while α_2_-AR agonist-induced inhibition of striatal GABA release is disinhibited in α_2C_-KO mice. Striatal ACh release may be inhibited in α_2C_-KO mice, indicating a role for the α_2C_-AR in mediating striatal acetylcholine release. **(B)** HVA concentrations are increased in the FC of α_2C_-OE mice. **(C)** Microdialysis assays show that treatment with the α_2C_-AR selective antagonist, ORM-10921, increases extracellular DA levels in the frontal cortex of Han-Wistar rats, while augmentation of a D_2_ antagonist with ORM-10921 increases brain-derived neurotrophic factor (BDNF) in striatal brain tissue of SIR rats. Further support that extends the relevance of these findings to mood/psychosis, and referred to elsewhere in the text, include reduced plasma corticosterone and antidepressant behaviors **(A)**; elevated plasma corticosterone and depressive behaviors **(B)**; and increased sensorimotor gating, improved cognition, and antipsychotic-like behaviors **(C)**. HVA, homovanillic acid; GABA, gamma-aminobutyric acid; KO, receptor knockout; OE, receptor overexpression; DA, dopamine; Ach, Acetylcholine; FC, frontal cortical; SIR, social isolation reared; Θ, inhibition.

The high expression of α_2C_-ARs in the striatum allows it to modulate presynaptic DA release and DA-mediated behaviors (26). Of particular interest is that Zhang and co-workers (64) provided early evidence for the ability of DA to function as an activating ligand on striatal α_2C_-ARs, while Sallinen and co-workers (18) used a novel α_2C_-AR selective antagonist (ORM-10921) to show increased *in vitro* α_2C_-AR potency and selectivity ratios in the presence of DA as agonist (Figure 2). These authors also reported that ORM-10921 increases extracellular DA levels in the rodent PFC. In support of the correlation between DA activity and α_2C_-AR activity, early studies indicated changes in brain DA metabolism in α_2C_-KO and α_2C_-OE mice (67) (Figure 3). α_2C_-OE mice show higher levels of the DA metabolite homovanillic acid (HVA) in the frontal cortex but not in the striatum compared to wild-type controls, whereas α_2C_-KO animals showed lower HVA concentrations in the striatum (67), although not in the frontal cortex (Figure 3). These findings suggest decreased striatal but not frontal cortical DA turnover in response to α_2C_-AR deactivation and increased cortical DA turnover in response to α_2C_-AR stimulation. Therefore, an important relationship exists between DA and the α_2C_-AR. The therapeutic potential of this can be realized in the targeting of α_2C_-ARs in disorders characterized by mesolimbic-cortical DA imbalance, such as schizophrenia or as demonstrated in SIR rats (20).

α_2C_-ARs also modify d-amphetamine-induced hyperlocomotion. Here d-amphetamine administration is associated with increased DA and NA release in the caudate nucleus and nucleus accumbens of the dorsal and ventral striatum, respectively, as well as in the PFC, together with co-presenting hyperactive behavior (70–72). Hyperlocomotion was further increased in α_2C_-KO mice following d-amphetamine administration, while d-amphetamine-induced hyperlocomotion was attenuated in α_2C_-OE mice (66). Subsequent studies with methylphenidate, a drug which also increases DA release and blocks DA and NA reuptake, showed increased response rates in a cognitive task sensitive to alterations in striatal DA levels in α_2C_-KO mice (73). The effects of drugs that increase synaptic DA could, therefore, be enhanced by antagonism of the α_2C_-AR, further emphasizing the role of α_2C_-ARs in regulating DA release and metabolism.

### Serotonin

Less evidence is available to delineate the role of the α_2C_-AR on serotonergic function. The hippocampal and cortical synthesis of the serotonin (5-HT) precursor, 5-hydroxytryptophan (5-HTP), *via* the rate-limiting enzyme tryptophan hydroxylase, seems to be dependent on both α_2A_-ARs and α_2C_-ARs in the rodent, with α_2A_-ARs emerging as the main α_2_-AR modulating 5-HT synthesis (31). Non-selective α_2_-AR agonism decreases 5-HTP levels in rodent hippocampus and cerebral cortex, while an increase in cortical 5-HTP levels seems to be largely induced by α_2A_-specific antagonism, with a α_2B/C_-AR antagonist producing an *increase* in 5-HTP levels (markedly less than that by a α_2A_-AR antagonist). These effects were not mirrored in the hippocampus, although α_2B/C_-AR specific antagonism decreased hippocampal 5-HTP levels in this brain region (31). Similarly, α_2_-AR-agonist-induced inhibition of 5-HT release is dependent on both α_2A_-ARs and α_2C_-ARs, although the α_2C_-AR exerts a more subtle effect on 5-HT release (33). These authors demonstrated that α_2C_-KO mice present with lower disinhibition of agonist-induced 5-HT release in hippocampal and occipito-parietal cortex slices compared to α_2A_-KO mice. The α_2A_-AR is, therefore, the main α_2_-AR regulating 5-HT release and possibly 5-HT synthesis. Nevertheless, selective antagonism of the α_2C_-AR could result in meaningful increases in 5-HT release and region-specific 5-HT synthesis (e.g., provoking serotonergic behaviours in Flinders Sensitive Line (FSL) rats (21)), which may be of importance in various neuropsychiatric illnesses characterized by altered serotonergic neurotransmission, such as obsessive compulsive disorder, MDD, and schizophrenia. Confirmation of these findings using highly selective α_2C_-AR subtype ligands and in appropriate animal models is, therefore, warranted (e.g., FSL rats; 21).

### Gamma-Aminobutyric Acid

Apart from effects on the synthesis and release of monoamines, the α_2C_-AR is an important mediator of striatal, but not hippocampal GABA release (65) *via* heteroreceptor actions. While α_2C_-ARs and α_2A_-ARs are located on different striatal neurons, almost all GABAergic projection neurons in the striatum contains α_2C_-ARs (60), which project to the globus pallidus and substantia nigra (74). Inhibition of striatal GABA release by an α_2_-AR antagonist (RX821002) is completely blocked in α_2C_-KO mice, while enhancement of striatal GABA release by an α_2_-AR agonist is maintained in these mice, suggesting that inhibition of striatal GABA release is strongly mediated by the α_2C_-AR (65). Striatal GABA’ergic transmission and response to α_2_-AC modulation is depicted in Figure 3. This response was not found with respect to hippocampal GABA release (65). These findings could suggest that selective blockade of the α_2C_-AR may mediate disinhibited GABA release in brain regions with dense dopaminergic innervation and low noradrenergic innervation (3). Considering the presence of α_2C_-ARs in the striatum (particularly the reward centers), and the role of GABAergic transmission in mania and the action of mood stabilizers (75), selective α_2C_-AR antagonism could be of value in disorders like schizophrenia in which deficient GABAergic transmission may play a pathophysiological role (76).

### Glutamate

Although it is known that α_2A_-AR modulate glutamate release *via* heteroreceptor-mediated cross-talk at glutamatergic neurons (77, 78), very little information is available on the specific role of the α_2C_-AR on central glutamatergic neurotransmission. Additional studies delineating the role of the α_2C_-AR on glutamatergic neurotransmission is warranted. Non-selective α_2_-AR antagonism *per se* does not seem to be beneficial in reversing NMDA-antagonist-induced cognitive impairment in rodent models (79), while non-selective α_2_-AR agonism may ameliorate these impairments (80–82). Contrasting the aforementioned findings, α_2C_-AR selective antagonists JP-1302, ORM-10921 and ORM-12741 reverse cognitive and social dysfunction in NMDA-antagonist-induced animal models of neuropsychiatric illness (17–19), indicating a beneficial role of selective α_2C_-AR antagonism (and *not* agonism) in attenuating symptoms induced by hypoglutamatergic states, although the mechanism is uncertain.

Disturbances in glutamate are well described in MDD and schizophrenia, while glutamatergic transmission represents an important target in pharmacological management of these disorders (68). Non-selective activation of α_2_ heteroreceptors on glutamatergic neurons by NA reduces glutamate release in various brain areas implicated in MDD and schizophrenia, including the frontal cortex, ventral tegmental area, hippocampus, and nucleus accumbens (77, 83). Moreover, the treatment arsenal for both MDD and schizophrenia include drugs that are α_2_-AR antagonists that would thus facilitate disinhibition of glutamate release. In support of this notion, the addition of a non-selective α_2_-AR-antagonist to a D_2_-blocker *increases* frontal cortical glutamatergic neurotransmission in rodents to a similar extent as the atypical antipsychotic clozapine, while at the same time improving cognitive and negative symptoms (3, 7, 84). Notably, clozapine has a threefold to fourfold higher α_2C_ over α_2A_ selectivity ratio and one of the highest α_2C_ over D_2_ selectivity ratios compared to other antipsychotics. The novel antipsychotic asenapine also presents with increased affinity for the α_2C_-AR and has good efficacy in treating both positive and negative symptoms of schizophrenia (4). Like that observed with clozapine and following the combination of a α_2_-AR lytic with a DA antagonist (7, 84), asenapine enhances frontal cortical glutamate transmission *via* DA activation of D_1_ receptors (85). Considering the above described effects of α_2_-lytic activity on prefrontal cortical glutamatergic transmission (84), measuring frontal cortical NMDA currents in NMDA-antagonist model of schizophrenia might elucidate the effects whereby α_2C_-AR selective antagonists improve NMDA-induced behavioral deficits.

Thus, the above findings suggest that α_2C_-AR antagonism allows the regulation of cortical glutamatergic transmission, which may underscore a therapeutic option in schizophrenia and cognitive dysfunction in particular. The involvement of α_2C_-ARs in the inhibition of striatal GABA release as mentioned above (65), could also indicate an indirect role of the α_2C_-AR in glutamate release, since glutamate release is also tonically regulated by GABAergic interneurons (86).

### Acetylcholine

Dysfunctional central cholinergic transmission has been implicated in the underlying pathophysiology of mood disorders, cognitive dysfunction, and schizophrenia [reviewed in Ref. (87)], while various drugs target the cholinergic system in an attempt at improving the above symptoms (88–90). Deficits in cholinergic transmission are also central to cognitive and memory dysfunction evident in Alzheimer’s disease (91). α_2_-adrenergic heteroreceptors, as well as D_2_ receptors, inhibit the release of acetylcholine (1). Similarly, the α_2C_-AR might be involved in the presynaptic regulation of cholinergic transmission. Since acetylcholine inhibits GABA release (92), Zhang and Ordway (65) have posited that α_2C_-AR effects on striatal GABA release (described above) might be attributed to the location of α_2C_-ARs on striatal cholinergic neurons. These authors have also reported that the α_2C_-AR mediates inhibition of striatal adenylyl cyclase and acetylcholine release, while these effects might be related to tonic activation of the α_2C_-AR by DA (64, 65). A selective α_2C_-AR antagonist might thus disinhibit striatal acetylcholine release that in turn may decrease extracellular striatal DA (87) (Figure 3). The findings of Zhang and Ordway (65) might thus be applicable to a neuropsychiatric disorder characterized by striatal dopaminergic over-activity, such as schizophrenia. A complex interplay of cortico-striatal cholinergic, GABAergic, and glutamatergic transmission has been described in the pathophysiology of schizophrenia (87), along with cholinergic regulation of dopaminergic and serotoninergic transmission and *vice versa*. However, more evidence in this regard using α_2C_-AR selective ligands is required to enable more definitive conclusions regarding the interplay of the α_2C_-AR, the cholinergic system and the effect of this interplay in neuropsychiatric disorders. Importantly though, the selective α_2C_-AR antagonist, ORM-12741, has demonstrated favorable effects on episodic memory in patients with Alzheimer’s disease (25), thus providing proof of concept regarding targeting of the α_2C_-AR in disorders of cognition, possibly *via* beneficial effects on cholinergic neurotransmission.

The α_2C_-AR thus seems to play a distinct role in monoaminergic, GABAergic, glutamatergic, and possibly cholinergic neurotransmission, making it a promising target in several neuropsychiatric illnesses characterized by dysregulation in the aforementioned pathways, in particular MDD, schizophrenia, and conditions associated with cognitive decline. The potential therapeutic role of the α_2C_-AR in these conditions, including an overview of evidence implicating its involvement in associated cognitive processes, will now be presented.

## Therapeutic Potential of Targeting the α_2C_-AR in MDD and Schizophrenia

### Behavioral Deficits Associated With MDD

A genetic polymorphism of the α_2C_-AR has been associated with emotional dysfunction in MDD (22). The α_2C_-AR is densely expressed in the hippocampus, an area that is prominent in the pathophysiology of MDD (93). MDD is thought to be characterized, at least in some patients, by deficits in monoamine activity and diminished inhibitory neural control of the hippocampus and PFC over the hypothalamic–adrenal–pituitary axis (HPA-axis), resulting in HPA-axis over-activity with reduced negative feedback and hypercortisolaemia (94). Additionally sleep alterations, deficient neurotrophic signaling and the effects of chronic stress on neurotrophic factors and hippocampal atrophy has been hypothesized to underlie the complex pathophysiology of the disorder (95, 96). Aside from limbic function, the hippocampus plays an important role in learning and memory, and hippocampal atrophy could account for the cognitive deficits that accompany MDD (93).

Antidepressants generally increase the levels of NA, 5-HT and DA to varying degrees depending on the class of antidepressant (97). However, about 40% of patients do not respond to the most commonly used conventional antidepressants (98, 99). Considering that α_2C_-ARs are densely expressed in the hippocampus, this AR subtype might be a potential target to address hippocampal-related disturbances in MDD. α_2_-AR dysregulation in depressive disorders is widely described in the literature [Ref. (46) for review], with increased α_2_-AR density found in platelets and in post-mortem brain tissue of depressed suicide completers in the locus coeruleus, temporal and frontal cortex, hippocampus and hypothalamus (100–103). Moreover, receptor upregulation has been specifically associated with the α_2A_-AR subtype in depressed states (104–106). The role of the α_2_-AR in the action of antidepressants is also fairly well described, of particular relevance being the α_2_-AR antagonist antidepressants, mirtazapine and mianserin (107, 108). Indeed, α_2_-AR downregulation is induced by tricyclic antidepressants (TCAs) and mirtazapine in rodents and depressed humans (brain and platelets), although regional differences in α_2_-AR downregulation have been noted in the CNS [reviewed in Ref. (46)].

The rodent forced swim test (FST) is a well-described predictive model for antidepressant drug screening (109, 110). In this test, rodents are exposed to inescapable swim stress where the adoption of an immobile posture during re-exposure is thought to reflect failure in persistent escape-directed behavior, purported to model certain behavioral aspects of MDD such as the psychological feeling of “entrapment” and the replacement of active coping strategies with passivity (109, 111, 112), also resembling avolition and despair noted in MDD. Specifically, an increase in immobility time is considered to reflect the aforementioned depressive-like manifestations. The majority of antidepressants reduce immobility time in the FST (109).

The α_2_-AR has been implicated in mediating the antidepressant (or anti-immobility) effects of TCAs in the FST, while activation of the α_2A_-AR subtype seems to be essential in this regard (41, 46). Interestingly, the α_2C_-AR plays an opposite role in regulating antidepressant effects in the FST. Early studies in α_2C_-OE models in mice have suggested that α_2C_-AR activation increases depressive behaviour in the FST, with α_2C_-OE mice displaying increased immobility compared to wild-type-controls (40) (Figure 3B), an effect not attributed to altered locomotor activity (67). On the other hand, α_2C_-KO mice demonstrate an antidepressive phenotype (40) (Figure 3A). These findings might explain why relatively non-selective α_2_-AR agonists (113–115) and certain non-selective α_2_-AR *antagonists* have both shown antidepressant-like effects in the FST. Recently these findings have been confirmed in rodents using subtype-selective α_2C_-AR antagonists. Acute administration of highly subtype-selective α_2C_-AR antagonists, JP-1302 (17), ORM-10921 (18, 21) and ORM-12741 (19) to Sprague Dawley and Han-Wistar rats was found to decrease immobility in the FST (see Table 2), providing evidence that selective α_2C_-AR antagonism harbors therapeutic antidepressant effects. Although the aforementioned findings were predominantly from acute studies, we recently reported that chronic ORM-10921 reduced FST immobility time in the FSL rat, a genetic rodent model of MDD (21). Moreover, these effects were not seen with the non-selective α_2_-AR antagonist idazoxan (21). These findings constitute the first findings for an antidepressant-like effect of an α_2C_-AR antagonist within a translational and pathological construct-driven approach (16). The beneficial effect of α_2A_-AR agonism on immobility in the FST as mentioned earlier and the increased immobility of α_2C_-OE mice observed in this test emphasizes that both the absence/minimization of α_2A_-AR antagonism and the presence of α_2C_-AR antagonism might be required for antidepressant-like effects. Earlier, we discussed how α_2A_-AR antagonism bolsters 5-HT transmission (33). Various studies have supported a therapeutic advantage for augmenting conventional antidepressants with α_2_-AR antagonists. Dhir and Kulkarni demonstrated potentiated anti-immobility effects in the FST when fluoxetine and venlafaxine are augmented with yohimbine (9). This effect is mirrored in the clinic, where the addition of yohimbine to selective serotonin reuptake inhibitor (SSRI) treatment hastens antidepressant response and increases the number of responders compared to SSRI treatment alone (116). Enhanced clinical response to SSRI’s, venlafaxine, and bupropion is also evident following augmentation with the α_2_-AR antagonist antidepressant mirtazapine, showing an early-onset of action (107, 117) as well as an almost doubling of the remission rate (118, 119). Clearly there is strong argument for adding an α_2_-AR antagonist to conventional antidepressant therapy.

**Table 2:** Neurochemical and behavioral findings in transgenic α_2C_-OE or α_2C_-KO mice, and data from rodent and human studies employing highly selective α_2C_-AR antagonists.

Parameter investigated	Findings in transgenic α_2C_-OE mice	Findings in transgenic α_2C_-KO mice	Findings in rodents and humans using highly selective α_2C_-AR antagonists
**Neurotransmission**			
Monoamine levels	α_2_-agonist-induced decreases in whole brain DA, NA, and 5-HT levels is absent in α_2C_-OE mice and OE-wt controls (67) Stress-induced elevations in whole brain HVA and 5-HIAA responses are attenuated in α_2C_-OE mice vs. OE-wt controls (40)	Increased levels of DA, NA, and 5-HT in whole brains of α_2C_-KO mice and KO-wt mice after treatment with α_2_-agonist (67) Stress-induced elevations in whole brain HVA and 5-HIAA in α_2C_-KO mice was similarly to KO-wt controls (40)	–

Dopamine turnover	Increased cortical DA turnover in α_2C_-OE mice (higher HVA levels) vs. OE-wt mice (67) Increased whole brain HVA levels in α_2C_-OE mice vs. OE-wt controls with a trend toward increased DOPAC (40)	Decreased striatal DA turnover in α_2C_-KO mice (lower HVA levels) vs. KO-wt mice (67) Decreased whole brain DOPAC and HVA concentrations in α_2C_-KO mice vs. KO-wt controls (40)	ORM-10921 increases extracellular DA in rodent prefrontal cortex (18)

Markers of neuronal activity	α_2C_-OE mice do not present with altered cortical and hippocampal levels of JunB and c-fos mRNA vs. OE-wt controls (40)	α_2C_-KO mice have increased cortical and hippocampal levels of JunB and c-fos mRNA vs. KO-wt controls. This difference disappears after stress (40)	–

Dopaminergic drug-induced hyperlocomotion	d-amphetamine-induced hyperlocomotion is attenuated in α_2C_-OE mice vs. OE-wt controls (66)	d-amphetamine-induced hyperlocomotion is further increased in α_2C_-KO mice vs. KO-wt controls (66)	–

Dopaminergic drug-induced cognitive reward responses	–	Increased response rates to methylphenidate in cognitive task sensitive to altered striatal DA in α_2C_-KO mice vs. KO-wt controls (73)	–

Striatal gamma-aminobutyric acid (GABA) release	–	α_2_-AR antagonist-induced inhibition of striatal GABA release is disinhibited in α_2C_-KO mice vs. KO-wt mice (65)	–

**Cognition**			
Working memory in MWM	α_2C_-OE mice show impaired navigation strategies vs. OE-wt controls Impaired navigation can be reversed by an α_2_-AR antagonist (19, 34–36)	–	ORM-12741 and ORM-10921 attenuates MK-801-disrupted learning in Sprague Dawley rats (18, 19)

Working memory in radial-arm maze	–	α_2_-AR agonist-induced working memory improvements are more pronounced in α_2C_-KO mice vs. KO-wt controls (37)	ORM-12741 attenuates PCP-disrupted working memory in Sprague Dawley rats (19) ORM-12741 attenuates age-related memory and learning deficits Sprague Dawley rats (19) ORM-12741 improves episodic memory in Alzheimer’s patients with a tendency to improve working memory (25)

Response learning in T-maze	–	α_2_-AR agonist does not induce improvements in response learning α_2C_-KO or KO-wt control mice, with no differences noted in drug naive α_2C_-KO vs. wt control mice (37)	–

Passive avoidance learning	α_2C_-OE mice show normal passive avoidance behavior vs. OE-wt controls (34)	–	–

**Depression**			
FST	Increased FST immobility time in α_2C_-OE mice vs. OE-wt mice (40)	Decreased FST immobility time in α_2C_-KO mice vs. KO-wt controls (40)	JP-1302 decreases FST immobility time in Sprague Dawley rats (17) ORM-12741 decreases FST immobility time in Sprague Dawley rats (19) ORM-10921 decreases FST immobility time in Sprague Dawley rats (18) ORM-10921 decreases FST immobility time in FSL rats (21)

Plasma corticosterone levels	Elevated stress-induced plasma corticosterone in α_2C_-OE mice vs. OE-wt controls after repeated, but not acute stress (40)	Attenuated stress-induced plasma corticosterone in α_2C_-KO mice vs. KO-wt controls (40)	–

Recognition memory in NORT	–	–	ORM-10921 improves object recognition memory (declarative memory) in the NORT in FSL rats (21)

**Schizophrenia**			
Sensory–motor gating	α_2C_-OE mice present with higher PPI vs. OE-wt controls (43)	α_2C_-KO mice present with deficient PPI vs. KO-wt controls (43)	JP-1302 reverses PCP-induced PPI deficits in Wistar and Sprague Dawley rats (17) ORM-12741 reverses PCP-induced PPI deficits in Sprague Dawley rats (19) ORM-10921 reverses SIR-induced PPI deficits in Sprague Dawley rats and augments the response to haloperidol on PPI to a similar extent as clozapine (20)

Social interaction	–	–	ORM-10921 and ORM-12741 attenuates PCP-induced social interaction deficits in Sprague Dawley rats (18, 19)

Recognition memory in NORT	–	–	ORM-10921 improves object recognition memory (declarative memory) in the NORT in SIR rats and augments the response to haloperidol to a similar extent as clozapine (20)

*DA, dopamine; DOPAC, 3,4-dihydroxyphenylacetic acid; 5-HT, serotonin; HVA, homovanillic acid; 5-HIAA, 5-hydroxy indole acetic acid; SIR, social isolation reared; MWM, Morris water maze; NA, noradrenaline; FST, forced swim test; NORT, novel object recognition test; FSL, Flinders sensitive line; PCP, phenylcyclidine; MK-801, dizolcipine; KO, receptor knockout; OE, receptor overexpression; wt, wild-type animals; PPI, prepulse inhibition test*.

The Novel Object Recognition Test (NORT) (see Cognitive Deficits Associated With MDD and Schizophrenia) measures recognition memory and is reliant on hippocampal function, while both this cognitive parameter (120, 121) and hippocampal function has been shown to be deficient in patients with MDD (93). Recently, an important role for the α_2C_-AR in this test has been demonstrated in the FSL rat, using the selective α_2C_-AR antagonist ORM-10921 in a chronic treatment paradigm (21). This study found that selective α_2C_-AR antagonism reversed deficits in novel object recognition memory in FSL rats, constituting the first findings for a pro-cognitive effect of a selective α_2C_-AR antagonist using an illness-specific construct-driven translational model of MDD.

Altered circadian rhythm is a well-recognized biomarker of MDD (68), with HPA-axis dysregulation and hypercortisolaemia underlying the pathophysiology of the disorder (94). Since stress and MDD are causally linked, stress-induced increases in glucocorticoids have been suggested to mediate hippocampal atrophy and neurodegeneration evident in depressed individuals (93, 122). This incapacitation of the hippocampus leads to impaired cognitive function as well as a perpetuation of the stress response, the latter due to an inability of the hippocampus to exert top-down control over the HPA-axis (122). Long-term exposure to elevated cortisol levels induces regional upregulation of α_2_-ARs (123), which in turn could result in further decreased NA levels. In this regard, the α_2_ antagonist and antidepressant mirtazapine has been associated with amelioration of HPA-axis hyperactivity in depressed patients (124, 125), albeit not necessarily related to clinical improvement. Interestingly, this amelioration of HPA-axis hyperactivity is not mirrored in rodents (126). In healthy volunteers, the acute administration of the α_2_-AR antagonist idazoxan has been associated with an attenuated normal diurnal fall in plasma cortisol, although dissipated following chronic treatment (127). Earlier studies on the other hand have shown that depressed patients exhibited much greater cortisol responses to yohimbine than controls (128). The α_2C_-KO mouse demonstrates attenuated plasma corticosterone elevations vs. wild-type controls following different stressors, while α_2C_-OE mice show more intense corticosterone responses compared to α_2C_-KO (40) (Figures 3A,B). Interestingly, non-selective α_2_-AR antagonism seems to elevate plasma corticosterone levels and to potentiate corticosterone responses to restraint stress in rodents (129). More selective α_2C_-AR antagonism might, therefore, elicit beneficial effects on HPA-axis functioning in depressive states. Previous studies have shown that both inhibition of corticosterone synthesis and injection of glucocorticoid receptor antisense oligonucleotides into the dentate gyrus of the hippocampus decreases immobility in the FST (130, 131). That the α_2C_-AR is the only α_2_-AR subtype expressed in this region in mice (67), together with the effects of α_2C_-AR modulation on corticosterone levels and FST immobility, consolidates a valuable role for α_2C_-AR antagonism in the treatment of MDD. Therefore, hypercortisolism in MDD may underscore a central dysfunctional adrenocortical feedback mechanism, with α_2_-ARs, and indeed the α_2C_-AR subtype specifically, being important in regulating glucocorticoid responses.

### Behavioral Deficits Associated With Schizophrenia

Associations between genetic polymorphism of the α_2C_-AR and certain aspects of psychotic disorders have been reported (24). Furthermore, α_2C_-ARs are most densely expressed in the striatum (132), where they are thought to play an inhibitory role (133). This dense expression has distinct importance when striatal dysfunction in schizophrenia is considered, especially its intricate connection to frontal cortical cognitive deficits (134). The α_2C_-AR, therefore, represents a potentially beneficial pharmacological approach to modulate striatal deficits in schizophrenia and possibly other psychotic disorders. The PFC, striatum and hippocampus are implicated in schizophrenia, where noradrenergic and dopaminergic terminals presenting with α_2C_ auto and heteroreceptors are well-represented (27, 59). Despite the prominence of the DA hypothesis of schizophrenia, a hypothesis implicating noradrenergic dysfunction also has significant support in the literature (135).

The DA paradox is well described in schizophrenia (136), with mesolimbic hyperdopaminergic and mesocortical hypodopaminergic states being postulated. Excessive striatal DA is linked to positive symptoms, while cognitive dysfunction is linked to deficits in cortical dopaminergic function (137). In Section “Role of the α_2C_-AR in Regulating Key Neurotransmitters,” we discussed findings that suggest decreased striatal but not frontal cortical DA turnover in response to α_2C_-AR deletion (Figures 3A,B), while increased cortical DA turnover has been noted in response to α_2C_-AR overexpression (67). These early findings suggest a positive role for α_2C_-AR antagonism in regulating mesolimbic-cortical dopaminergic imbalances, which may have therapeutic value in schizophrenia. GABAergic and glutamatergic deficits are also implicated in schizophrenia disease pathology, where loss of GABAergic output onto secondary glutamatergic cortical neurons required for tonic control over subcortical dopaminergic neurons, results in increased mesolimbic dopaminergic firing (increased striatal DA release) and consequently the presentation of psychotic symptoms (86). As discussed earlier, the α_2C_-AR strongly mediates striatal GABA release, while α_2C_-AR deletion seems to disinhibit α_2_-AR antagonist-induced inhibition of GABA release (65). Here, α_2C_-AR subtype-selective antagonism might present with more beneficial effects on striatal GABA release than non-selective α_2_-AR antagonism when applied as pharmacological treatment of schizophrenia.

**Figure 4:**
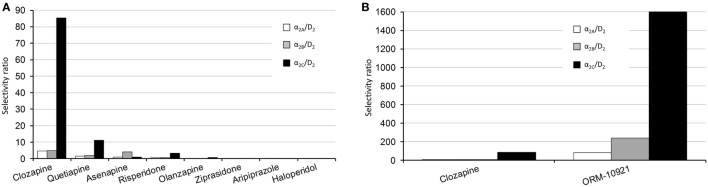
Human α_2_-AR subtype/D_2_ selectivity ratios of various antipsychotics, adapted from Ref. (4). **(A)** Left panel, comparative overview of the subtype selectivities of various antipsychotics. The α_2C_/D_2_ receptor selectivity ratios are as follows: clozapine, 85; quetiapine, 11; risperidone, 3.4; asenapine, 1; olanzapine, 0.53; ziprasidone, 0.02; haloperidol, 0.011. **(B)** Right panel, comparison between the subtype-selective ratios of clozapine and the α_2C_-AR antagonist ORM-10921, which has shown antipsychotic-like effects in preclinical studies (18, 20). The α_2C_/D_2_ receptor selectivity ratio for ORM-10921 is 1,600. Selectivity ratios were determined by dividing the D_2_ K_i_ value by the applicable α_2_ receptor K_i_ value.

The atypicality of antipsychotic drugs primarily reflects their reduced risk of extra-pyramidal side effects and to some extent improved efficacy against negative and cognitive symptoms of schizophrenia (138), over and above their efficacy against positive symptoms. Atypicality has, apart from actions at serotonergic receptors, been proposed to revolve around α-AR modulation, with α_1_ and α_2_-AR antagonism suggested to contribute to stabilization of dysregulated dopaminergic activity (139). Indeed, in a thorough comparative study employing human receptor binding data, Shahid and colleagues (4) have shown that a number of atypical antipsychotics (clozapine, quetiapine, asenapine, risperidone, ziprasidone) possess significant α_2_-AR antagonist properties. Furthermore, quetiapine and in particular clozapine showed prominent α_2C_ over D_2_ as well as α_2C_ over α_2A_ receptor selectivity. A pharmacological profile constituting a higher α_2_ vs. D_2_ receptor binding ratio (139), and specifically a higher α_2C_ vs. D_2_ receptor selectivity ratio (3, 4), has been suggested to mediate the improved efficacy of drugs like clozapine that exhibit lower D_2_-receptor occupancy. The α_2_ over D_2_ receptor subtype selectivity ratios for various antipsychotics as well as the α_2C_-AR selective antagonist, ORM-10921 (which as described below has shown antipsychotic-like activity in animal models), are depicted in Figures 4A,B. Thus, reduced D_2_-receptor occupancy might be possible in therapy because of the beneficial effects of additional α_2_-AR antagonism on dysregulated dopaminergic activity, allowing for improved efficacy with less motor side effects. Support for this hypothesis has been demonstrated in studies employing non-selective α_2_-AR antagonists (e.g., idazoxan) as augmentation to D_2_-receptor antagonist antipsychotic treatment (7, 84, 140). While this combination of α_2_-AR and D_2_ receptor antagonism presents with improved antipsychotic-like effects in mouse models of schizophrenia, it also resulted in enhanced cortical glutamatergic transmission and increased dopaminergic output in the PFC, with subsequent improvement in cognitive parameters in rats (84). The effects of this augmentation strategy were comparable to that of clozapine. While clozapine requires approximately 45% D_2_ receptor occupancy compared to >70% required by other D_2_ receptor antagonists for antipsychotic efficacy (141, 142), the combination of idazoxan with a D_2_ receptor antagonist exhibited potent antipsychotic effects similar to that of clozapine at similar low D_2_ receptor occupancy rates (84).

Sensorimotor gating refers to the ability to integrate and process sensorimotor information, deficits of which are suggested to underlie the fragmentation of reality evident in schizophrenia (143). The prepulse inhibition (PPI) of startle test refers to the attenuation of a startle response produced by the presentation of a smaller prepulse, and is used to study the gating of sensorimotor information by the brain (143, 144). A typical example of the PPI test in humans employs the somatosensory eye blink reflex in response to acoustic, tactile (e.g., air puffs) or light stimuli (143, 145, 146). A PPI deficit can be induced in humans and animals by various psychotomimetic drugs, including dopaminergic and antiglutamatergic drugs. Animal models of schizophrenia, such as SIR (147–149) and various transgenic models including mice with altered DA, 5-HT, and glutamate receptor expression (150), present with deficits in PPI. Importantly, antipsychotic drugs normalize disrupted PPI in animals and humans (151–155). While the contribution of non-selective α_2_-blockade to modulation of PPI has been proposed, the literature is somewhat inconclusive in this regard. In fact, some papers have suggested that antagonism of the α_2A_-AR does not contribute to enhancement of the PPI (44, 156–158).

Considering the important role for α_2_-AR antagonism in managing schizophrenia (139), earlier studies in transgenic mouse models have demonstrated that antipsychotic-like effects are subtype dependent. In this regard, α_2C_-KO mice demonstrated clear PPI deficits compared to wild-type controls, while α_2C_-OE mice had markedly higher PPI scores than their wild-type controls (43), suggesting that α_2C_-receptor agonism may induce antipsychotic-like effects. However, this extrapolation from transgenic mouse studies has since been disproven following experiments with selective α_2C_-AR antagonists. JP-1302, ORM-10921, and ORM-12741 that consistently show improved PPI in Sprague Dawley and Wistar rats in NMDA-antagonist-induced models of schizophrenia (17–19). More recent findings in SIR rats, a putative neurodevelopmental model of schizophrenia (159, 160), corroborate these earlier findings, with ORM-10921 found to significantly improve SIR-associated PPI deficits in a manner comparable to clozapine (20) (Figure 3C). Moreover, ORM-10921 also enhanced the effects of haloperidol on the above-mentioned deficits in PPI (20). Earlier, in Section “Distinct Roles for α_2_-AR Subtypes,”, we highlighted this discrepancy, emphasizing the need to corroborate findings from transgenic mouse models with studies employing subtype-selective ligands in suitable animal models.

Cognitive deficits in schizophrenia make up some of the core elements of the disorder (161) and are often refractory to treatment (162). These impairments include deficits in working, recognition and spatial memory, cognitive flexibility, learning, and attention (163–165). However, antipsychotic treatments are not always reproducibly effective in reversing these cognitive deficits in animal models (166–170), which in fact reflects the relative lack of efficacy displayed by antipsychotics in treating cognitive impairment in the clinic (164, 165). Recently, the highly selective α_2C_-AR antagonist ORM-12741 showed improved effects on NMDA-antagonist-induced disruptions in working memory and spatial learning, navigation and memory in rodents (19). NMDA-antagonist models include the administration of the glutamate NMDA-receptor antagonists dizolcilpine (MK-801) or PCP which are known to induce behavioral, cognitive, and neurochemical disruptions in behavior akin to those seen in schizophrenia (171). ORM-12741 attenuates the disruption of learning in the Morris Water Maze (MWM) induced by MK-801, while also improving PCP-induced memory deficits in the 8-arm radial maze (19). Similar findings were reported for the selective α_2C_-AR antagonist ORM-10921 which attenuates MK-801-induced spatial navigation in the MWM (18), a finding consistent with effects described for atypical (167, 172) but not typical antipsychotics such as haloperidol (173). Additionally, ORM-10921 significantly improved object recognition memory in SIR rats, comparable to the atypical antipsychotic clozapine, while also significantly improving the efficacy of haloperidol in this regard (20) (Figure 3C). Evidence of improved cognition in NMDA-antagonist and neurodevelopmental models of schizophrenia with novel highly selective α_2C_-AR antagonists, therefore, demonstrates the therapeutic potential of targeting the α_2C_-AR in treating cognitive deficits associated with schizophrenia.

Another interesting observation concerns the neurotrophic hypothesis of schizophrenia, where reduced brain-derived neurotrophic factor (BDNF) is widely evident in the illness (174, 175), as well as being associated with the above-mentioned cognitive deficits (68). Although chronic treatment with the α_2C_-AR antagonist, ORM-10921, alone did not significantly reverse lowered BDNF levels in SIR rats on its own, combining haloperidol with ORM-10921 showed a significant increase in BDNF levels that exceeded that of either drug alone (20) (Figure 3C). These preliminary results further support a therapeutic role for α_2C_-AR antagonism in improving cognitive symptoms in schizophrenia.

Social isolation, decreased social cognition, and impaired social skills form part of the negative symptoms of schizophrenia and are refractory to most antipsychotic treatments (176). The social interaction test measures deficits in social motivation and self-directed behavior in rats and is used to measure predictive validity of antipsychotics in rodent models of schizophrenia (177). Although there are mixed results, generally atypical antipsychotics are more effective than typical antipsychotics at attenuating social deficits in these models (177, 178). In this regard, the α_2C_-AR antagonists ORM-10921 and ORM-12741 significantly attenuate PCP-induced deficits in social interaction in short-term single-housed and pair-housed rats (18, 19).

Since especially atypical antipsychotics have activity at the α_2C_-AR, it is important to consider data from functional assays on these compounds using cloned receptors in Chinese hamster ovary cell lines. Kalkman and Loetscher (3) found α_2C_ over α_2A_ receptor selectivity ratios for clozapine, chlorpromazine, risperidone, quetiapine, and iloperidone to be between 3 and 12, indicating that most atypical antipsychotics present with higher α_2C_-AR antagonist activity than α_2A_-AR antagonist activity. Additionally, the novel antipsychotics asenapine and lurasidone both present with potent α_2C_-AR binding affinity (4, 179). α_2C_ over D_2_ selectivity ratio has, therefore, been suggested to be an important factor in antipsychotic efficacy (3). Clozapine, arguably the most efficacious antipsychotic in treatment refractory schizophrenia (180), presents with an α_2C_ over D_2_ selectivity ratio of 85 compared to ratios of 0.01–11 for other tested antipsychotics (4) (see Figure 4A). Haloperidol, on the other hand, has the lowest potency at the α_2C_-AR as well as the lowest α_2C_ over D_2_ ratio of tested compounds (3, 4), and is not regarded as an atypical antipsychotic. However, bolstering its antipsychotic-like and pro-cognitive effects with a selective α_2C_-AR-antagonist (20) supports the notion that an increased α_2C_ over D_2_ ratio will translate to superior antipsychotic effects. Taken together, α_2C_-AR antagonism is involved in the mechanism of improved sensorimotor gating, cognitive, and social functioning in pharmacological and neurodevelopmental models of schizophrenia. These data are indicative of a therapeutic role for α_2C_-AR antagonism in the treatment of schizophrenia, and further study with more subtype-selective ligands is encouraged.

### Cognitive Deficits Associated With MDD and Schizophrenia

Many neuropsychiatric illnesses, including MDD and schizophrenia, present with cognitive deficits and memory impairments (122, 162, 163, 165). The α_2C_-AR has been shown to be involved in cognitive deficits evident in both non-pathological (34–37) and pathological (20, 21) animal models, with findings implicating a significant role in the treatment of cognitive deficits associated with these disorders. Although α_2_-AR agonists are associated with improved cognitive processing in humans and animals (38, 181–184) and in the treatment of cognitive decline associated with aging (185), these effects have been shown to be mediated *via* activation of the α_2A_-AR (37–39), which is also responsible for sedative and hypotensive effects (51, 186). In contrast, genetic deletion of the α_2C_-AR subtype, or by extrapolation selective α_2C_-AR antagonism, has been demonstrated to improve memory and cognition in the MWM, the 8-arm radial maze and the NORT, as discussed below. Furthermore, α_2C_-AR antagonism has been found to benefit neurotrophins and other biomarkers of neuronal resilience associated with cognition (20).

The MWM is a spatial water navigation task requiring the rodent to learn and remember the location of an escape platform in a water arena in order to locate a hidden (submerged) platform in subsequent trials by using various spatial cues. The escape latency is a measure of spatial working memory. The test is a reliable tool correlating with hippocampal synaptic plasticity as well as intact glutamate NMDA-receptor function (187). In early transgenic mouse studies, α_2C_-OE mice showed impaired spatial and non-spatial escape strategies and search patterns in the MWM. Administration of an α_2_-AR antagonist could reverse these impairments to a greater extent in α_2C_-OE than in wild-type mice, suggesting that α_2C_-AR antagonism might play a more prominent role than α_2A_-AR antagonism in brain areas involved in spatial navigation (34–36). Considering the dense expression of the α_2C_-AR in the hippocampus and striatum and that hippocampal (188) and striatal lesions (189) impair aspects of MWM navigation, α_2C_-AR selective antagonism may mediate information processing and behavioral adaptation to environmental change. α_2C_-OE mice display normal passive avoidance learning, suggesting that impaired water maze navigation in α_2C_-OE mice does not reflect defective stimulus-response learning and that α_2C_-AR deactivation is, therefore, associated with the processes underlying complex organization of escape behavior (34). This effect of α_2C_-AR antagonism might partially explain previous findings for pro-cognitive effects of the non-selective α_2_-AR antagonist, idazoxan, on planning, attention, episodic memory and verbal fluency in patients with frontal lobe dysfunction (11).

The radial-arm maze is a test used to measure reference and working memory in rodents and relies on intact functioning of the prefrontal cortical, hippocampal and striatal interconnections to locate food rewards hidden in various radial-arm target sites (190). Björklund and co-workers (37) demonstrated that the non-selective α_2_-AR agonist dexmedetomidine improves working memory in the radial-arm maze, and that this improvement is greater in α_2C_-KO mice, suggesting that the absence of α_2C_-AR agonism (or simultaneous α_2C_-AR antagonism) might result in enhanced performance with respect to working memory.

The NORT is a two-trial behavioral measure that relies on the rodent’s innate preference to explore novel objects over familiar objects, thereby enabling measurement of recognition memory (191, 192). The declarative memory processes underlying the NORT relies on the perirhinal cortex and the hippocampal complex (193–195). Uys and colleagues have demonstrated that selective α_2C_-AR antagonism with ORM-10921 markedly improves recognition memory in pathological animal models of schizophrenia (20) and MDD (21).

The above-mentioned benefits of selective α_2C_-AR antagonism on cognitive parameters have been corroborated with studies employing highly selective α_2C_-AR antagonists in animal models of schizophrenia, MDD, and age-related cognitive impairment (18–21), as described in Sections “Behavioural Deficits Associated With MDD” and “Behavioural Deficits Associated With Schizophrenia,” as well as in clinical trials investigating novel therapy for Alzheimer’s disease (25) (see Evidence for Targeting the α_2C_-AR in Other Neuropsychiatric Disorders).

Brain-derived neurotrophic factor is the most prevalent neurotrophic growth factor in the CNS where it is especially important in regulating synaptic plasticity and various aspects underlying cognitive performance, memory, and mood (196, 197). Acute and chronic stress purportedly have detrimental effects on rodent BDNF expression in the hippocampus, while altered BDNF levels are evident in depressive disorders (68, 198) and in schizophrenia (174, 175). While both antipsychotics and antidepressants alter BDNF levels to varying degrees (199–203), non-selective α_2_-AR antagonism has also been associated with neurogenesis and increased BDNF levels in the hippocampus (204, 205). Noradrenergic (202, 206), dopaminergic (207), serotonergic (208), and GABA-glutamate (209) interactions are involved in the expression of BDNF. With the α_2C_-AR acting as a heteroreceptor to modulate the release of many of the aforementioned neurotransmitters, this receptor might play an indirect role in regulating the expression of BDNF. Evidence for the involvement of the α_2C_-AR in the expression of BDNF has been demonstrated in the SIR animal model of schizophrenia, where SIR rats present with *reduced* striatal BDNF levels (20). While conventional antidopaminergic antipsychotics are not associated with correction of said reduced BDNF levels (201), a recent study reported that combining haloperidol with the selective α_2C_-AR antagonist ORM-10921 (but not α_2C_-AR antagonism *per se*) increases striatal BDNF levels in these animals, while at the same time improving deficits in cognition and sensorimotor gating (20). This study indicated that not only is augmentation with a α_2C_-AR antagonist associated with improved BDNF expression but also that this improvement is correlated with improved cognitive performance, thus supporting a role for α_2C_-AR antagonism in disorders associated with reduced cognitive flexibility and deficits in neurotrophin support.

Brain-derived neurotrophic factor is also important in regulating C-fos and JunB expression, biomarkers of neuronal activity that play an important role in synaptic function (210, 211). Upregulation of c-fos mRNA is induced by noxious stimuli, neurotransmitters, neurotrophins and other growth factors as well as learning and memory processes (212). Jun-B is also involved in the regulation of emotional memory (213). BDNF restores the expression of these transcription factors after neuronal insult (214), reinforcing BDNF’s role in neuroplasticity at gene-transcription level. Interestingly, cortical and hippocampal levels of c-fos and JunB mRNA are increased in α_2C_-KO mice compared to wild-type controls (40), while this is not the case in α_2C_-OE mice. Whether this increase is associated with altered BDNF levels in α_2C_-KO mice has not been investigated. Nevertheless, the increase in neuronal activity in α_2C_-KO mice is of interest considering the pro-cognitive behavioral characteristics of this transgenic strain and the above-mentioned beneficial effects of α_2C_-AR antagonists on BDNF expression and cognitive performance.

Thus, antagonism of the α_2C_-AR might benefit cognitive processes relevant to both MDD and schizophrenia. Since cognitive deficits are core symptoms of both disorders, the α_2C_-AR related effects on cognition and neuronal markers of plasticity support the therapeutic potential of targeting the α_2C_-AR in these disorders.

To summarize findings from transgenic mouse models and those gained from treatment with α_2C_-subtype-selective ligands, Table 2 presents neurochemical and behavioral findings reported in transgenic mice and in various rodent models predicting pro-cognitive-like, antidepressant-like and antipsychotic-like effects as described in Sections “Behavioural Deficits Associated With MDD,” “Behavioural Deficits Associated With Schizophrenia,” and “Cognitive Deficits Associated With MDD and Schizophrenia”. As a GPCR that functions within the PSD, the synaptic actions of the α_2C_-AR and indeed drugs that target this receptor, might involve regulatory PSD proteins to mediate the aforementioned effects.

### Putative Involvement of PSD Proteins

The PSD is a specialized matrix located at excitatory postsynaptic terminals, described as a macromolecular complex of several hundreds of proteins that act as a molecular switch for multiple interacting neurotransmitter signaling pathways (215). Such proteins include those containing the PSD-95/disc large/zonula occludens-1 (PDZ) domain, and the membrane-associated guanylyl kinase, all of which comprise three PDZ peptide-binding domains (215). These proteins in turn promote binding to a variety of molecules within the PSD necessary for signal transduction (45). We have earlier noted the importance of the PSD in postsynaptic GPCR signaling. There is significant interest in the role of the PSD network in psychopharmacology and psychotropic drug action, although much of the extant evidence in support of this relates to DA and glutamate-dependent synaptic plasticity (45, 215). Nevertheless, this review has highlighted the importance of heteroreceptor-directed modulation of DA and glutamate signaling by the α_2C_-AR, while at least one prominent PDZ-domain binding protein, spinophilin, has been associated with the α_2_-AR (45). Spinophilin regulates α_2_-AR associated G_αi_ coupling, membrane localization, endocytosis, receptor desensitization and calcium signaling (216–218). Despite this evidence, however, spinophilin is not yet known to be involved in major neuropsychiatric disorders or to by modulated by main psychopharmacologic treatments (215). Nevertheless, it is perhaps worth discussing how and why a PSD protein such as spinophilin may mediate important pharmacological responses following ligand binding to the α_2C_-AR.

Although the specific role for PSD proteins in psychiatric illness remains speculative, clinical and preclinical studies have provided evidence for their involvement in aberrant synaptic plasticity [see Ref. (215) for review]. These processes are invariably associated with high-order cognitive alterations, which are essentially the core pathophysiology in a number of psychiatric diseases, including depression and schizophrenia (219–221).

When considering a therapeutic strategy in psychiatric diseases, psychotropic-mediated modulation of PSD molecules may occur either directly or indirectly, the latter as a consequence of drug interaction with their target non-PSD receptors. Currently, there is no known agent that directly targets a PSD protein for therapeutic effect. Since PSD molecules are modulated by antipsychotics and antidepressants (221–226), as well as play a key role in behavioral response (227, 228), they represent putative targets for pharmacological action. Moreover, concurrent administration of antipsychotics and antidepressants may induce synergistic modulation of specific PSD molecules (229–231), which provides at least conceptual support for targeting the PSD to address treatment resistance in mood and psychotic disorders. This is particularly relevant when discussing the α_2_-AR, since a number of studies have described enhanced efficacy of typical and atypical antipsychotic drugs by adjunctive α_2_-AR blockade (20, 84, 232). Concerning the α_2C_-AR, the α_2C_-AR antagonist, ORM-10921 has been found to bolster the response to haloperidol in social isolation reared rats at both the level of synaptic plasticity (i.e., BDNF) and cognition (i.e., object recognition memory) (20). That the combined response was similar to clozapine emphasizes the benefit of adjunctive α_2C_-AR antagonism with regard to treatment response. Such data holds promise for application in treatment resistance, and further studies in this regard, but combined with co-assessment of PSD proteins, are warranted.

This review has focused on the therapeutic potential of targeting the α_2C_-AR subtype in MDD and schizophrenia. However, cognitive dysfunction is common in patients with Alzheimer’s disease, MDD and schizophrenia, while symptoms of the latter two illnesses permeate through to patients suffering from Alzheimer’s disease. Indeed, recent preclinical and preliminary clinical evidence has revealed the promising therapeutic role for the α_2C_-AR in addressing cognitive decline in Alzheimer’s disease. Co-presentation of cognitive decline in this and other disorders, and the role of the α_2C_-AR, warrants brief discussion.

## Evidence for Targeting the α_2C_-AR in other Neuropsychiatric Disorders

ORM-12741 is a novel highly selective α_2C_-AR antagonist with a 4000-fold selectivity for α_2C_-AR vs. the α_2A/B_-AR (19). Age-related memory and learning, as assessed in the rodent 8-arm radial maze (measuring spatial working memory and reference memory), was attenuated by sub-chronic administration of ORM-12741 (19), and more recently confirmed in a phase IIa randomized, double-blind, placebo-controlled clinical study in patients with moderate Alzheimer’s disease (25). Here, ORM-12741 was used as an add-on drug in patients already receiving donepezil, galantamine, rivastigmine or memantine. Significant improvements in episodic memory were observed, as well as a tendency to improve working memory. In addition, ORM-12741 produced significant improvement in perceived levels of distress with respect to symptoms of delusions, agitation and aggression, MDD, anxiety, disinhibition and other behavioral symptoms (25). These findings are not unlike the augmentation data described in preclinical studies with another α_2C_-AR antagonist, ORM-10921 (18). Moreover, there was a positive trend to lower caretaker distress scores which would also reflect reduced symptom severity and frequency (25). Thus improvements in cognitive performance in Alzheimer’s disease are supported by amelioration of co-presenting behavioral impairments, of which some are reminiscent of those presenting in MDD and schizophrenia.

Although the beneficial role of α_2_-AR agonism in strengthening prefrontal cortical function and enhancing working memory has been described in ADHD, these affects are associated with postsynaptic stimulation of the α_2A_-AR subtype (47). Early evidence has, however, also suggested a potential therapeutic role for selective targeting of the α_2C_-AR subtype in ADHD. A study in coloboma mice, a mouse model of ADHD (233), reported that the α_2C_-subtype preferring α_2_-AR antagonist MK912 (~10-fold selectivity over α_2A_-AR and α_2B_-AR) ameliorated NA-dependent hyperactivity (234), while α_2A_-AR and α_2B_-AR subtype-preferring drugs were ineffective. Considering the pronounced expression of the α_2C_-ARs in the basal ganglia, the authors suggest that α_2C_-AR antagonism might be a useful treatment for locomotor-related and hyperactivity functions in coloboma mice and by implication a potential therapeutic target for ADHD. It is conceivable that part of the mode of action of a selective α_2C_-AR antagonist may involve indirectly facilitating activation of postsynaptic α-AR including α_2A_-AR as a consequence of increase in synaptic NA. These effects need to be corroborated using subtype-selective ligands with higher selectivity ratios, and subsequent testing on cognition in models of ADHD.

A comment on the role of the α_2C_-AR in bipolar disorder is also warranted. Bipolar disorder is a mood disorder characterized by mixed symptoms of MDD and mania, with both antidepressants (235) and antipsychotics (236) in combination with mood stabilizers advocated as standard first-line treatment. Quetiapine is an atypical antipsychotic with a favorable α_2C_/α_2A_ and a fairly high α_2C_/D_2_ ratio (3, 4) (see Figure 4A) that has shown marked clinical efficacy in treating mania and MDD in bipolar disorder (236, 237). In the light of evidence provided in the afore going sections, future studies investigating the therapeutic potential of targeting the α_2C_-AR in bipolar disorder using α_2C_-AR selective ligands could provide valuable insights.

Finally, given the prominent role of NA in the neurobiology and treatment of anxiety and fear-related manifestations (238), exploratory studies into the use of α_2C_-AR antagonists in anxiety disorders are also encouraged.

## Future Perspective: What Do We have and What Do We Need?

Recent developments and the current state of knowledge support the therapeutic potential of selectively targeting the α_2C_-AR in the treatment of MDD, schizophrenia and associated cognitive dysfunction. Antidepressant and antipsychotic treatment benefits are likely to include broader/enhanced efficacy (e.g., facilitation of postsynaptic cortical α_2A_-AR activity) as well as reduced side effects (e.g., liability for cardiovascular effects). There is, however, limited clinical data in this respect and further patient trials are urgently needed. In addition, despite recent progress there are still significant gaps in the knowledge base relating to the function, physiology, and pharmacology of α_2C_-ARs. Some areas requiring further research include:

- α_2C_-AR signal transduction pathways and trafficking in brain tissue from normal and disease model animals.
- Assessing treatment response following combined α_2C_-AR antagonism with a typical/atypical antipsychotic or antidepressant, using an animal model of treatment resistance, e.g., Ref. (239, 240), and combining with co-assessment of PSD proteins.
- Assess the effect of α_2C_-AR, alone or in combination with an antipsychotic/antidepressant, on the expression of PSD proteins, such as spinophilin, PSD-95, etc.
- α_2C_-AR receptor regulation; differences in human disease tissue or animal models, and whether existing treatments, e.g., for schizophrenia and MDD, alter α_2C_-AR density.
- Distribution and cellular localization of α_2C_-ARs at noradrenergic and non-adrenergic synapses and whether these receptors play an extra-synaptic role.
- Heteroreceptor function and mode of modulation of non-adrenergic neurotransmitter release, particularly in the hippocampus and frontal cortex.
- Insight on putative receptors (e.g., 5-HT_1A_, D_1_, AMPA receptors) that may be involved in mediating the *in vivo* central effects of selective α_2C_-AR receptor antagonism.
- Contribution toward modulation of stress and inflammation-linked pathways.
- Evaluation in animal models with strong disease construct (e.g., genetic, age, stress) and applying more translationally relevant approaches (e.g., chronic treatment, gender differences, altered circadian rhythms, augmentation strategies).
- Further experimental studies with new imaging tools [e.g., positron emission tomography (PET) ligand ORM-13070] to establish the role of the α_2C-_AR in the brain of healthy subjects and patients.
- Considering the high comorbidity of anxiety in these illnesses, and that it can significantly affect prognosis and treatment response (241, 242), to study the anxiolytic capabilities of α_2C_-AR modulators in appropriate models.

An array of tools is now available to facilitate further research. Highly selective α_2C_-AR subtype ligands, and specifically α_2C_-AR selective antagonists, have been developed over the past decade. Before that, drugs with marginal selectivity were used to delineate pharmacological effects of the α_2_-AR subtypes. For example, although BMY7378 is mainly an α_1D_-AR antagonist, it also presents with a 10-fold selectivity for α_2C_-ARs vs. α_2A_-ARs (243). Another example of an antagonist drug with marginal α_2C_-AR selectivity is MK912, which also displays approximate 10-fold greater selectivity for α_2C_-ARs vs. α_2A_-AR and α_2B_-ARs (57, 58) and has been used to delineate the role of the α_2C_-AR on hyperactive behavior in a mouse model of ADHD (234).

In 2008, *N*-{2-[4-(2,3-dihydro-benzo[1,4]dioxin-2-ylmethyl)-[1,4]diazepan-1-yl]-ethyl}-2-phenoxy-nicotinamide was synthesized, and found to display >100-fold selectivity for α_2C_-AR vs. α_2A_-AR, with excellent binding affinity and functional activity at the α_2C_-AR in rats. Although low selectivity vs. α_2B_-ARs was shown, the α_2B_-ARs have negligible distribution in the CNS. This compound displayed excellent binding affinity and functional activity for α_2C_-ARs in rats, with adequate CNS penetration (244). Further animal studies with this promising compound are eagerly awaited.

In 2007, Orion Pharma reported that their novel selective α_2C_-AR antagonist, JP-1302, presented with a minimum 50-fold selectivity for the α_2C_-AR with an α_2C/2A_ ratio of 93 (17). However, this compound does not optimally enter the CNS. In 2013 another Orion Pharma compound with improved CNS penetration, ORM-10921, was characterized with an α_2C/2A_ ratio of ~100 in rodents, although this ratio was found to be lower in human cells (~29) (18). Both JP-1302 and ORM-10921 have since been used safely in preclinical studies in rodent models of neuropsychiatric illness and highlighted in this review. On the other hand, the novel α_2C_-AR antagonist, ORM-12741, has been tested for safety and efficacy in both rodents and humans (19, 25) and presents with a 4000-fold selectivity for the α_2C_-AR vs. α_2A_-AR and α_2B_-AR. This highly selective α_2C_-AR antagonist is currently in clinical trials for the treatment of symptoms associated with Alzheimer’s disease (25).

An important recent development has been the development of ORM-13070, a selective α_2C_-AR which is amenable to labeling with ^11^C and has been successfully used as a _α2C_-AR PET tracer that readily enters the CNS (245). This compound has a binding affinity selectivity of over 200-fold vs. the α_2A_-AR, with weak or no activity at more than 100 other potential target sites and receptors, and will be highly valuable for facilitating forward and reverse translation between animal and human studies. An obvious application is determination of target engagement, through conducting receptor occupancy studies for novel drug candidate molecules for preclinical and clinical studies (62, 245, 246). However, it could also be used to gain more precise insight on the relative _α2C_-AR occupancy for antipsychotic (e.g., clozapine) and antidepressant (e.g., mirtazapine) agents at clinical doses thus enabling a better understanding on the mode of action of these drugs. The tracer could also be of potential value to investigate disease-related changes in receptor density and effects on neurotransmitter activity. The latter aspect has been investigated and in line with evidence that the _α2C_-AR is sensitive to low synaptic concentrations of NA, ^[11C]^ORM-13070 shows increased CNS binding in response to decreased synaptic NA (132).

On the other side of the spectrum, novel α_2C_-AR agonists have also been characterized recently. [*N*-[3,4-dihydro-4-(1H-imidazol-4-ylmethyl)-2H-1,4-benzoxazin-6-yl]-*N*-ethyl-*N*′-methylurea] or “Compound A” and a chemically similar “Compound B” were found to be highly selective for the α_2C_-AR, albeit with poor brain penetration. These compounds are being investigated for effects on peripheral vasoconstriction (245, 247).

With the aim to stimulate further investigation into the value of the α_2C_-AR in neuropsychiatric disorders, genetic and molecular biology driven approaches will also be critical. Mice overexpressing or lacking the α_2C_-AR have been generated but have been phenotyped to a limited extent. Further behavioral and biological characterization, for example using -omics type molecular profiling, as well as regionally restricted genetic manipulation using genetic deletion technology in rats, would yield valuable data. The zebrafish is another platform of discovery that may provide a powerful model in which to study developmental and genetic factors that underlie human disease (248). Work in zebrafish has shown that the zebrafish α_2_-AR subtypes are markedly conserved compared to mammalian α_2_-AR subtypes with similar pharmacological profiles and functional effects compared to human α_2_-AR subtypes (249, 250). This model might also be beneficial in future studies when characterizing novel subtype-selective α_2_-AR ligands.

## Conclusion

This review has provided an overview of recent developments and future direction in research investigating the role of the α_2C_-AR in neuropsychiatric illness and therapy, with specific focus on the effects of α_2C_-AR antagonism in cognition, MDD, and schizophrenia. Targeting this receptor could present with beneficial therapeutic effects and decreased noradrenergic side effects when used alone or as augmentation strategy in the treatment of these diseases, as well as disorders presenting more specifically with cognitive decline, such as Alzheimer’s disease. The recent advent of clinical grade subtype-selective α_2C_-AR antagonists has contributed toward delineating the neuropsychopharmacology of this receptor. Studies employing these novel highly selective α_2C_-AR ligands in putative translationally relevant animal models of psychiatric illness, to inform further experimental medicine evaluation in humans, will be vital in strengthening our understanding of the α_2C_-AR as a therapeutic target.

## Author Contributions

MU prepared the first draft of the manuscript, prepared all the figures and tables, as well as managed all subsequent changes and formatting. MS reviewed the manuscript and provided input on the manuscript design and content, as well as on the figures and tables. BH was the study leader and student supervisor to MU, developed the article concept and design, and finalized the manuscript for submission.

## Conflict of Interest Statement

MS is an employee of Orion Pharma. No funding was received by Orion Pharma for this, or previous work by these authors. The authors declare that over the past 3 years, BH has participated in advisory boards and received honoraria from Servier^®^, and has received research funding from Servier^®^ and Lundbeck^®^. ORM-10921, which was used in recent studies by the authors and cited in this paper was sponsored by Orion Pharma. BH declares that, except for income from the primary employer and research funding from the below-mentioned organizations and agencies, no financial support or compensation has been received from any individual or corporate entity over the past 3 years for research or professional services, and there are no personal financial holdings that could be perceived as constituting a potential conflict of interest. The authors declare no other conflicts of interest.

## Abbreviations

ADHD, attention-deficit hyperactivity disorder; AR, adrenoceptor; BDNF, brain-derived neurotrophic factor; cAMP, cyclic adenosine monophosphate; CNS, central nervous system; DOPA, 3,4-dihydroxyphenylalanine; DA, dopamine; FST, forced swim test; FSL, Flinders Sensitive Line; FRL, Flinders resistant line; GABA, gamma-aminobutyric acid; HPA-axis, hypothalamic–pituitary–adrenal axis; HVA, homovanilic acid; KO, knockout; MAPK, mitogen-activated protein kinase; MK-801, dizolcipine; MWM, Morris Water Maze; NA, noradrenaline; NMDA, *N*-methyl-d-aspartate; NORT, novel object recognition test; OE, overexpressing; PCP, phenylcyclidine; PPI, prepulse inhibition; PSD, postsynaptic density; SIR, social isolation reared/social isolation rearing; SSRI, selective serotonin reuptake inhibitor; TCA, tricyclic antidepressant; 5-HIAA, 5-hydroxyindoleacetic acid; 5-HT, serotonin; 5-HTP, 5-hydroxytryptophan.
